# Folate, vitamin B12 and vitamin D status in healthy and active home-dwelling people over 70 years

**DOI:** 10.1186/s12877-023-04391-2

**Published:** 2023-10-18

**Authors:** Felix Kerlikowsky, Jan Philipp Schuchardt, Andreas Hahn

**Affiliations:** Institute of Food Science and Human Nutrition, Leibniz University Hannover, Hannover, Germany

**Keywords:** Nutrient status, HoloTC, RBC Folate, Ageing

## Abstract

**Background:**

Ageing is characterised by physiological changes that can affect the nutrient availability and requirements. In particular, the status of vitamin D, cobalamin and folate has often been found to be critical in older people living in residential care. However, there is a lack of studies investigating the status of these nutrients in healthy and active home-dwelling elderly people.

**Methods:**

The aim of this cross-sectional study was to assess the status of vitamin D based on serum concentrations of 25-hydroxycholecalciferol [25-(OH)D], cobalamin based on serum concentrations of holotranscobalamin (holoTC) and folate based on red blood cell (RBC) folate in unsupplemented, healthy and active German home-dwelling subjects ≥ 70 years of age (n = 134, mean ± SD: 75.8 ± 4.5 years). Dietary intake was assessed by 3-day food recalls. The study was conducted between March and November of 2021 (during the COVID-19 pandemic).

**Results:**

The mean 25-(OH)D concentration was high at 85.1 ± 26.0 nmol/L, while the majority of women (92%) and men (94%) had 25-(OH)D concentrations ≥ 50 nmol/L. Less than 10% of men and women had 25-(OH)D concentrations < 50 nmol/L. The mean holoTC concentration was 88.9 ± 33.7 pmol/L (94.8 ± 34.6 pmol/L in women and 73.6 ± 25.6 in men). Only 8% of the women were cobalamin deficient (< 50 pmol/L holoTC) compared to 22% of the men. The mean RBC folate concentration was 831 ± 244 nmol/L, while the prevalence of folate deficiency was 10%. Linear regression analysis showed that only folate equivalent intake was associated with the relevant nutrient status marker.

**Conclusion:**

Our findings suggest that healthy, independently living older people with high levels of education, physical activity, and health awareness are not necessarily at higher risk of vitamin D, folate and cobalamin deficiency. Further studies are needed to verify these findings and to identify lifestyle and dietary patterns that can predict adequate nutrient status for healthy ageing.

**Trial registration:**

This study is officially recorded in the German Clinical Trials Register (DRKS00021302).

**Supplementary Information:**

The online version contains supplementary material available at 10.1186/s12877-023-04391-2.

## Introduction

The supply of nutrients such as vitamin D, cobalamin and folate is sometimes critical for the general population in many countries around the world [1]. Older people in particular are at risk of insufficient intake or deficiency of these nutrients due to age-related dysfunction (e.g., reduced mucosal integrity) or an unbalanced diet [2]. This is supported by the fact that diet-related metabolic disorders, such as type 2 diabetes or cognitive and neuromuscular dysfunction, increase with age [1, 3].

1,25-dihydroxyvitamin D, the active form of vitamin D, is important not only for bone and tooth formation, but also for the immune system and the neuropsychiatric function [4]. The primary determinant of vitamin D status is not dietary intake, but endogenous synthesis, which may be insufficient due to reduced sun exposure during the winter months and decreased capacity with age. In conclusion, the minimum serum 25-(OH)D level can be observed in February and March, and the maximum in late summer [5].

There is an ongoing debate about the target concentrations of circulating 25-(OH)D needed to maintain health, and suggested cut-off values in scientific publications and advisory bodies vary [6]. The National Academy of Medicine (NAM) and European Food Safety Authority (EFSA) consider 25-(OH)D concentrations > 50 nmol/L to be sufficient for metabolic health [7, 8]. For the prevention of falls and fractures, some guidelines also recommend 25-(OH)D concentrations > 75 nmol/L as desirable, which is particularly important for older people with advanced degenerative bone resorption processes [9, 10].

The supply of cobalamin may be critical for the elderly due to its complex absorption process, which may be affected by age-related disorders [11–13]. Dietary intake may be sufficient for older omnivores, but reduced absorption due to atrophic gastritis as well as *Helicobacter pylori* infection, chronic use of proton pump inhibitors or lack of intrinsic factor may lead to deficits in cobalamin status [14]. Because clinical symptoms such as macrocytic anaemia and neuropathy are not immediately apparent, cobalamin deficiency often goes unrecognised for a long time [12]. It is therefore important to screen also apparently healthy elderly individuals for cobalamin deficiency using valid long-term markers such as holotranscobalamin (holoTC) [15].

The main reason for folate deficiency is an unbalanced diet that is low in unprocessed vegetables, whole grains, and legumes, or vitamin losses during meal preparation. In the general population the dietary intake of folate (equivalents) is often below the recommendations [16, 17], which can lead to inadequate folate status. In addition, age-related changes lead to a decreased sense of hunger, while satiety signals become faster and stronger [2]. In conclusion, despite a good health state, the frequency of meals and the total amount of food consumed may decrease in older people. In addition, older people avoid eating raw and unprocessed fruits and vegetables. Instead, intense heating and gentle cooking make it easier to consume fruits and vegetables, but reduce the bioavailability of folate from foods. Folate deficiency can lead to elevated homocysteine levels, which are associated with a higher risk of cardiovascular disease and cognitive decline in older people [18, 19].

There is a lack of data on the vitamin D, cobalamin, and folate status in older but otherwise healthy older people, especially those aged 70 years and older living independently. The associations between education, physical activity, health attitudes and awareness, alcohol consumption, smoking, and medications on the one hand and the vitamin D, cobalamin, and folate status on the other hand in the elderly population are rarely investigated. As a result, it is difficult to draw conclusions about the nutritional status of at-risk groups such as the elderly. In addition, nutritional randomised controlled trials (RCT) in older people tend to reach those who already have a sophisticated understanding of health maintenance and disease prevention, making it difficult to draw conclusions about the population as a whole.

Therefore, the primary aim of our study was to evaluate the vitamin D, cobalamin, and folate status in unsupplemented, healthy, independently living elderly people ≥ 70 years of age using reliable, state-of-the-art status markers. The secondary aim of the study was to investigate associations of the vitamin status markers with the age and intake of specific dietary food groups.

## Materials and methods

### Study design and participants

The cross-sectional evaluation was performed using baseline data from a larger study. The original study was a randomized, double-blind, placebo-controlled trial involving 134 subjects aged ≥ 70 years with the overall aim of assessing and improving the status of critical nutrients in older people. Sample size was calculated using an expected drop-out rate of 10%, a significance level of 5%, and a power of 80%. To detect differences in the two-sided t-test between the verum and placebo groups with a Cohen’s effect size of 0.5, a case number of 60 subjects per group (n = 120 in total) was obtained. Details of this study have been reported elsewhere [20].

Briefly, the baseline data collection was conducted in accordance with the guidelines of the Declaration of Helsinki and carried out at the Institute of Food Science and Human Nutrition in Hannover, Germany (hereinafter referred to as the “Institute”) between March 2021 and November 2021. The ethic committee of the medical chamber of Lower Saxony (Hannover, Germany) approved all procedures. Written informed consent was obtained from all participants prior to their enrolment.

**Fig. 1:**
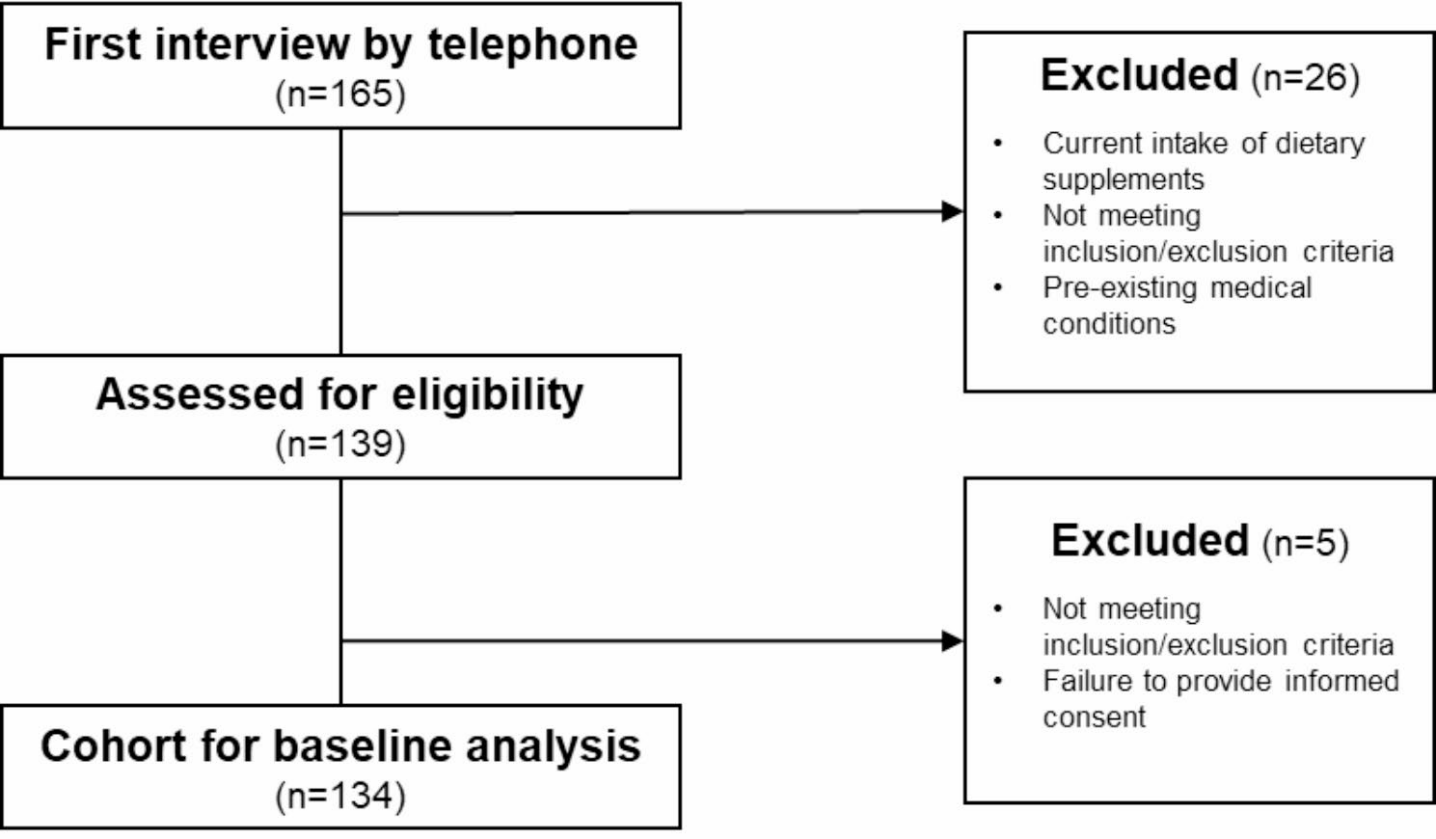
Flow chart of study population

Participants were recruited by announcing the study in the local press, at senior networking centres and volunteer clubs. All interested participants were screened for their health status and controlled for inclusion and exclusion criteria, 134 subjects were invited to the Institute for examination by Institute’s staff members **(**Fig. 1**).** The main inclusion criteria were an age ≥ 70 years and an independently, home-dwelling living situation. Exclusion criteria were defined as current cardiovascular, metabolic or malignant disease as well as current or up to three months past use of dietary supplements. All interested subjects were asked by telephone about their use of dietary supplements before being invited to the study. If they did not use any supplements, they were invited to the study day, where they were asked a second time about their use of supplements in the previous three months. In case of a conflict of interest, interested subjects were excluded from the study.

### Food and nutrient intake

Participants completed 3-day food recalls, including two consecutive weekdays and one weekend day. The PRODI6.4® dietary software based on the German Federal Food Code 3.02 (Nutri-Science GmbH, Freiburg, Germany) was used to analyse the amount of food, food groups and nutrient-specific data such as energy, macronutrients, minerals and vitamins in the reported diet over three days. The 3-day food recall information was also used to assess the consumption of fortified and/or energy-reduced foods. The dietary questionnaires were scored by trained nutritionists of the Institute. Food and food group intakes were reported and calculated based on energy adjustment using the residuum method previously described by Willet et al. [21].

### Lifestyle and health behaviour

A questionnaire on medical history, current medication use (frequency and dosage), health status and attitude, selected questions on dietary, and physical activity was filled out by all study participants. The following classification was chosen to describe the physical activity behaviour: “predominantly active” (> 2 ½ h/week of moderate-intensity or > 1 ¼ h/week of vigorous-intensity exercise); “predominantly sedentary” (< 2 ½ h/week of moderate-intensity or < 1 ¼ h/week of vigorous-intensity exercise); or “regular exercise” (approximately 2 ½ h/week of moderate-intensity or 1 ¼ h/week of vigorous-intensity exercise).

Alcohol consumption was assessed using 3-day food recalls (see above). The maximum acceptable level for women and men was set at 10 g and 20 g of pure alcohol per day, respectively, according to [22]. To characterise their “attitudes to health”, subjects were asked whether they expected their health to get worse. Subjects were classified as having an “optimistic self-perception of ageing” or a “pessimistic self-perception of ageing”. The exact use of medical drugs was recorded using a specific case report form questionnaire. Within this questionnaire the frequency of use and daily dose were recorded in a free-text response.

### Anthropometric and body composition measurements

Height was measured using a stadiometer (Seca GmbH & Co. KG, Hamburg, Germany). Waist circumference (WC) was measured between the lowest rib and the highest hip bone at the narrowest part of the midsection using a tape measure. Body weight was measured digitally (Seca GmbH & Co. KG, Hamburg, Germany) to the nearest 0.1 kg (lightly dressed, without shoes). The body composition markers fat mass (FM), lean body mass (BLM), total body water (TBW) and phase angle (PA) were analysed using an 8-point bioelectrical impedance analyser (BIA, mBCA525, Seca Company, Hamburg, Germany). For the measurements, participants were instructed to urinate and remove all jewellery before the examination. Subjects then had to lie down on a stretcher and rest for about 5 min to ensure a balanced distribution of body fluids. All measurements were taken by trained nutritionists of the Institute.

### Blood sampling and blood pressure measurement

After an overnight fast (≥ 12 h fasting period), blood samples were collected by a physician between 08:00 and 11:00 a.m. Blood samples were taken by venipuncture from an arm vein using multifly needles (Sarstedt, Nümbrecht, Germany) into serum or EDTA monovettes (Sarstedt). All samples were stored at ~ 5 °C and shipped to external laboratories on the same day. Blood pressure was measured by the physician in the sitting position after a resting period of 3–5 min on both upper arms above the elbow.

### Biochemical analysis of 25-(OH)D, holoTC and RBC folate

Serum 25-(OH)D was measured in duplicate at SYNLAB MVZ (Leinfelden, Germany) using liquid chromatography coupled to tandem mass spectrometry (LC-MS/MS, Recipe, Munich, Germany). For serum 25-(OH)D analysis, mean recoveries were obtained between 85 and 104% with a within-assay coefficient of variation of 2.8% and a limit of detection (LOD) of 1.61 nmol/L and a limit of quantification (LOQ) of 5.4 nmol/L. Serum holoTC was determined using electrochemiluminescence immunoassay (ECLIA) on cobas® test systems (Roche Diagnostics GmbH, Mannheim, Germany) as previously described [15, 23]. For holoTC, mean recoveries were obtained between 88 and 106% with a within-assay coefficient of variation of 3.4% and a limit of detection (LOD) of 3.0 pmol/L and a limit of quantification (LOQ) of 5.0 pmol/L. RBC folate was analysed using ECLIA on Immulite 2000 analyser series (Diagnostic Products Corporation, Los Angeles, USA) with a mean recoveries between 98 and 104%, analytical sensitivity of 1.8 nmol/L and within-assay coefficient of variation of 4.5%.

Concentrations of vitamins are reported in nmol/L in case of RBC folate and 25-(OH)D and in pmol/L in case of holoTC concentrations. Creatinine concentrations are reportd in µmol/l and CRP concentrations in mg/dL. All numbers were rounded to three significant digits.

### Reference ranges

In agreement with the recommendations from the NAM and the EFSA the cut-off for serum 25-(OH)D concentrations with > 50 nmol/L, as indicative for a “sufficient” vitamin D status, was applied [7, 8]. 25-(OH)D concentrations between 25 - <50 nmol/L were classified as “insufficient” and concentrations < 25 nmol/L as “deficient” according to the classification of numerous recent publications [6, 24–26]. For “cobalamin deficiency”, a holoTC concentration cut-off of < 50 pmol/L was applied [27]. Reference concentrations for RBC folate are highly dependent on the laboratory-specific assay used. With the RBC folate method used by SYNLAB MVZ (Leinfelden, Germany), the reference range of 570–1810 nmol/L was specified. Thus, RBC folate concentrations of < 570 nmol/L indicate a “folate deficiency” in the current study population.

### Data analysis and statistical methods

All analysis were performed using SPSS statistical software (version 28.0; SPSS, Chicago, IL, USA). The Shapiro-Wilk test was used to test the normal distribution of 25-(OH)D, holoTC and RBC folate concentrations. In addition, quantile-quantile plots were generated for visual inspection. In case of absence, log transformation was performed to obtain a normal distribution. Multiple linear regression models were used to examine associations between concentrations of markers of nutrient status and age and food group intakes. Only food groups considered to be relevant sources of vitamin D, cobalamin and folate were included in the regression analysis. Model 1 represents unadjusted regression analysis and model 2 was adjusted for age and sex and model 3 was fully adjusted for total energy intake, age, sex, body weight, BMI, WC and creatinine concentrations. Statistical significance was set at the level of 0.05.

## Results

### Baseline characteristics

In total, 134 elderly people were included in the study (Table 1). The age ranged from 70 years up to 100 years at the timepoint of examination with a mean age of 75.8 ± 4.5 years. Almost the same number of subjects lived alone or within a partnership at home (49.4% vs. 50.6%).

Mean body weight, BMI, WC, and body composition markers were within the physiologically range for healthy elderly subjects. Mean serum creatinine concentrations of women (70.4 ± 8.8 µmol/l) and men (88.0 ± 17.7 µmol/l ) were within the laboratory specific reference ranges (women: 44.9–83.6 µmol/l; men: 59.0-103 µmol/l). Mean plasma CRP concentrations were also in a very low range.

The cohort was characterised by a high level of education (54% high education level and 15% low education level) compared to the generel German population aged 65–80 years (14% high education level and 56% low education level) [28].

21% of subjects reported not taking any medication, but 12% had polyvalent medication use (≥ 5 medications at the same time).

The majority of the subjects were omnivores. 13% of the subjects reported being vegetarians.

Only 10% of the subjects described their physical activity as predominantly sedentary and more than 95% of the subjects were current non-smokers. In addition, about 70% of this cohort reported no alcohol consumption or alcohol consumption below the maximum acceptable level. Finally, participants’ attitudes to health were predominantly optimistic, with 66% feeling positive about their future health.

**Table 1:** Anthropometric and demographic characteristics, lifestyle and health behaviour of the study population

	Total n = 134	Female n = 97	Male n = 37
	Mean ± SD	Mean ± SD	Mean ± SD
Anthropometrics			
Weight [kg] BMI [kg/m^2^] WC [cm] FM [%] BLM [%] TBW [L] PA [°] Creatinine [µmol/L] CRP [mg//dL]	67.4 ± 13.1 25.7 ± 4.6 92.6 ± 12.2 35.6 ± 8.9 64.4 ± 9.0 33.6 ± 7.3 4.9 ± 0.5 79.2 ± 17.6 2.2 ± 0.9	67.4 ± 13.1 25.6 ± 5.0 90.4 ± 12.4 38.6 ± 7.9 61.4 ± 7.9 30.7 ± 5.6 4.8 ± 0.5 70.4 ± 8.8 2.3 ± 0.9	78.0 ± 12.7 25.8 ± 3.1 98.2 ± 9.9 27.8 ± 6.4 72.1 ± 6.4 41.2 ± 5.5 5.1 ± 0.6 88.0 ± 17.7 1.9 ± 1.1
	**n (%)**	**n (%)**	**n (%)**
**Age groups**			
70–74 years 75–79 years ≥ 80 years	61 (46) 51 (38) 22 (16)	50 (52) 35 (36) 12 (12)	11 (30) 16 (43) 10 (27)
**Family status**			
Living alone Living with a partner	65 (49) 68 (51)	59 (62) 37 (38)	6 (16) 31 (84)
**Education level**			
Low education Middle education High education	20 (15) 41 (31) 72 (54)	16 (17) 34 (35) 46 (48)	4 (11) 7 (19) 26 (70)
**Medical drug intake** ^a^			
No intake Antihypertensive drug Statins Proton-pump inhibitor Polypharmacy	30 (21) 72 (53) 25 (18) 9 (7) 16 (12)	24 (25) 49 (51) 10 (10) 6 (6) 8 (8)	6 (16) 23 (62) 15 (41) 3 (8) 8 (22)
**Usual diet**			
Omnivor Vegetarian	114 (87) 17 (13)	80 (81) 15 (19)	34 (94) 2 (6)
**Physical activity** ^b^			
Predominantly active Predominantly sendentary Regular basis movement	25 (19) 13 (10) 96 (71)	20 (21) 10 (10) 67 (69)	5 (14) 3 (8) 29 (78)
**Smoking status**			
Current smoker Previous smoker^c^ Never smoke	3 (4) 60 (46) 67 (50)	3 (5) 43 (46) 48 (49)	0 (-) 17 (49) 19 (51)
**Alcohol use** ^d^			
Abstinent < maximum acceptable level > maximum acceptable level	33 (25) 62 (46) 39 (29)	25 (26) 45 (46) 27 (28)	8 (22) 17 (46) 12 (32)
**Attitudes to health**			
Optimistic self-perception of ageing Pessimistic self-perception of ageing	85 (66) 43 (34)	61 (67) 30 (33)	24 (65) 13 (35)

BMI: body mass index; WC: waist circumference; FM: fat mass; BLM: body lean mass; TBW: total body water; PA: phase angle. ^a^ Physical activity: predominantly active > 2½ h/week of moderate-intensity or > 1¼ h/week of vigorous-intensity exercise; predominantly sedentary < 2½ h/week of moderate-intensity or < 1¼ h/week of vigorous-intensity exercise or regular exercise approximately 2½ h/week of moderate-intensity or 1¼ h/week of vigorous-intensity exercise. ^b^ Previous smoker: at least one year without smoking. ^c^ Maximum acceptable level of alcohol use: women: 10 g/day; men: 20 g/day according to [29]. ^d^ Multiple answers possible. Polypharmacy: ≥5 medical drugs at same time

### Vitamin D intake and 25-(OH)D concentrations

The dietary intake of vitamin D was low, with a mean ± SD of 4.1 ± 5.0 µg/day in the entire study group. The serum 25-(OH)D concentration of the entire study population was 85.1 ± 26.0 nmol/L (Table 2). The majority of study subjects had 25-(OH)D concentrations ≥ 50 nmol/L, indicating a sufficient vitamin D status (93%). Less than 10% of the participants in this study had an inadequate or deficient vitamin D status during the season of maximum UVB radiation. Using linear regression analyses, we found no significant association between vitamin D intake and serum 25-(OH)D concentration (Table 3).

### Cobalamin intake and holoTC concentrations

The dietary intake of cobalamin in the entire study population was 4.0 ± 2.1 µg/day. The holoTC concentration of the entire study population was 88.9 ± 33.7 pmol/L (Table 2). The prevalence of cobalamin deficiency was low in women but relatively high in men. Only 8% of the women had a deficient cobalamin status compared to 22% of the men. The intake of cobalamin was not significantly associated with holoTC concentrations (Table 3).

### Folate intake and RBC folate concentrations

The dietary intake of folate equivalents was 251 ± 95.6 µg/day in the total study population. The RBC folate concentration of the entire study population was 831 ± 244 nmol/L (Table 2). Women had slightly higher RBC folate concentrations than men (845 ± 256 nmol/L vs. 795 ± 210 nmol/L). The prevalence of folate deficiency in the entire group was low (10%), with minor differences between the sexes. Folate equivalent intake was significantly associated with RBC folate concentrations in a fully adjusted model (Table 3, Model 3, p = 0.002).

**Table 2:** Blood concentrations of holotranscobalamin [holoTC], red blood cell [RBC] folate and 25-hydroxycholecalciferol [25-(OH)D]

	Total n = 134	Female n = 97	Male n = 37
	Mean ± SD *n (%)*	Mean ± SD *n (%)*	Mean ± SD *n (%)*
**HoloTC** [pmol/L] Deficiency [< 50 pmol/L]	88.9 ± 33.7 *16 (12)*	94.8 ± 34.6 *8 (8)*	73.6 ± 25.6 *8 (22)*
**RBC folate** [nmol/L] Deficiency [< 570 nmol/L]	831 ± 244 *13 (10)*	845 ± 256 *9 (10)*	795 ± 210 *4 (12)*
**25-(OH)D [nmol/L]**	85.1 ± 26.0	85.9 ± 26.8	83.1 ± 23.8
Deficiency [≤ 25 nmol/L] Insufficiency [25 - <50 nmol/L]	*2 (1)* *8 (6)*	*1 (1)* *7 (7)*	*1 (3)* *1 (3)*
Sufficiency [≥ 50 nmol/L]	*124 (93)*	*89 (92)*	*35 (94)*

**Table 3:** Association of dietary cobalamin, folic acid and vitamin D intake and vitamin status markers

	Beta-coeff. Model 1		p-value Model 1	Beta-coeff. Model 2	p-value Model 2	Beta-coeff. Model 3	p-value Model 3
**HoloTC**		0.024	0.151	0.073	0.188	0.095	0.243
**RBC folate**		0.001	0.062	0.013	0.057	0.057	**0.002**
**25-(OH)D**		0.001	0.716	0.002	0.679	0.062	0.237

Model 1: Unadjusted using cobalamin intake as an independent variable for holoTC, folic acid intake as an independent variable for RBC folate and vitamin D as an independent variable for 25-(OH)D. Model 2: Adjusted for age and sex. Model 3: Adjusted for age, sex, energy intake, body weight, BMI, WC and creatinine

### Association between dietary food group intake and vitamin status markers

Consumption of milk and dairy products (Table 4) was significantly associated with holoTC concentrations in the unadjusted (p < 0.001) and fully adjusted (p < 0.001) model (Table 5). Similarly, vegetable intake (Table 4) was significantly associated with RBC folate concentrations in the unadjusted (p = 0.005) and the sex and age-adjusted model (p = 0.044), but not in the fully adjusted model (p = 0.268). For all other food groups, no associations with the vitamin status markers were found.

**Table 4:** Energy-adjusted dietary food group intake calculated via 3-day food recalls

	Total n = 134	Female n = 97	Male n = 37
	Mean ± SD	Mean ± SD	Mean ± SD
**Food group intake**			
**Fruits** [g/day]	188 ± 127	192 ± 127	177 ± 124
**Vegetables** [g/day]	247 ± 148	255 ± 140	225 ± 166
**Seeds and nuts** [g/day]	14.1 ± 19.4	15.9 ± 20.4	9.26 ± 15.7
**Fish** [g/day]	33.9 ± 36.0	35.0 ± 38.4	30.9 ± 36.0
**Milk/ dairy products** [g/day]	154 ± 116	162 ± 100	132 ± 149
**Meat, eggs, meat products** [g/day]	82.9 ± 62.3	77.1 ± 59.1	98.7 ± 69.7
**Grains and bread [g/day]**	118 ± 54.1	110 ± 52.7	137 ± 54.1

The energy adjustment was performed using the residuum method, as previously described by Willet et al. [21]

**Table 5:** Association of food group intake and vitamin status markers

	Beta-coeff. Model 1		p-value Model 1	Beta-coeff. Model 2	p-value Model 2	Beta-coeff. Model 3	p-value Model 3
**HoloTC**							
Milk and dairy products		0.235	**< 0.001**	0.148	**< 0.001**	0.173	**< 0.001**
Meat, eggs, meat products		0.001	0.236	0.054	0.537	0.052	0.675
**RBC folate**							
Fruits		0.222	0.300	0.011	0.590	0.041	0.865
Vegetables		0.498	**0.005**	0.053	**0.044**	0.020	0.268
Seed and nuts		0.341	0.135	0.020	0.179	0.016	0.605
Grains and bread		0.002	0.961	0.018	0.728	0.052	0.949
**25-(OH)D**							
**Fish**		0.017	0.787	0.008	0.545	0.031	0.172

Model 1: Unadjusted. Modell 2: Adjusted for age and sex. Modell 3: Adjusted for age, sex, energy intake, body weight, BMI, WC and creatinine

## Discussion

The aim of the present study was to assess vitamin D, cobalamin, and folate status in unsupplemented, healthy, independently living, active elderly people aged ≥ 70 years.

Although we expected a better status of these nutrients compared to the general population, we were surprised by the very low prevalence of vitamin D, cobalamin and folate deficiencies. Only the male subjects showed a slightly higher prevalence of cobalamin deficiency. However, since the number of men in the study was very small, this result should be treated with caution. Comparable studies in healthy subjects aged ≥ 70 years using tissue markers such as holoTC and RBC folate are rare. In addition, several of these studies included cohorts with a high prevalence of regular use of supplements or fortified foods in countries where this is applicable, which is in contrast to our cohort of unsupplemented individuals.

### Vitamin D status

The prevalence of vitamin D deficiency has been investigated in many epidemiologic studies in Europe, with 25-(OH)D being the most commonly used marker [6, 30, 31]. The studies differed in the analytical methods used to investigate serum 25-(OH)D concentrations, with the LC/MS-MS technique being considered the gold standard compared to the chemiluminescent microparticle immunoassay (CLIA). In our study, serum was measured in duplicate by LC/MS-MS, so the results should be analytically accurate.

Several epidemiological studies have shown that the prevalence of 25-(OH)D concentrations < 50 nmol/L is between 30% and 80% worldwide at all ages [32]. Previous observations examining vitamin D status, particularly in older adults ranged from 16 to 79%, depending on season and sex [5, 6, 33, 34]. In the KORA Age Study [33], a cross-sectional study in southern Germany (age range 65 to 93 years, n = 1,079), the prevalence of vitamin D insufficiency was 52%. In comparison, the prevalence of vitamin D insufficiency in the present cohort was low at less than 10%. Our results are also in contrast with data from the German Nationwide Nutrition Survey 1 (DEGS1), which included 6,995 individuals of all ages and reported a high prevalence of vitamin D insufficiency during summer time. Specifically, about half of the adults of both sexes had a vitamin D insufficiency [22]. It should be noted that serum 25-(OH)D in the DEGS1 study was analysed by the CLIA method, which has been described to yield falsely low levels of 25-(OH)D compared to the LC-MS/MS method as used in our study [35]. Under this assumption, the difference in 25-(OH)D concentrations to our data may be reduced. Klenk et al. [5] also observed that the prevalence of 25-(OH)D concentrations < 50 nmol/L in a population of elderly subjects (≥ 65 years, not taking supplements, measured in August) in southern Germany was 16.1% (mean 25-(OH)D: 77.6 nmol/L), which is in line with our results.

The present cohort was characterised by a high level of physical activity, mainly outdoors during the COVID-19 pandemic, resulting in high exposure to UVB radiation, which is the strongest factor influencing the vitamin D status [22, 36, 37]. In addition, the population had a low average BMI and body fat percentage compared to those reported in the DEGS1 study [38]. Therefore, it’s important to emphasise that this sample size is not representative of the entire elderly population in Germany.

Dietary sources usually cover up only 10–20% of the vitamin D requirement and therefore do not significantly influence the vitamin D status [39, 40]. This is also evident in our study, where we did not find significant associations between vitamin D intake and 25-(OH)D concentrations. However, a Dutch study as part of the B-PROF trial in 2,530 people aged ≥ 65 years showed a significant association between vitamin D intake and 25-(OH)D concentrations during the summer period [41]. Within a dose-response relationship, the authors suggest a 1 nmol/L increase in 25-(OH)D concentrations with each unit increase in vitamin D intake.

### Cobalamin status

HoloTC reflects long-term cobalamin intake and is considered the most valid marker to assess cobalamin status, especially in older individuals (> 50 years) [15]. We observed a prevalence of cobalamin deficiency (12%) in the entire study population. Especially men showed a high prevalence of cobalamin deficiency with 22%. The sex differences in cobalamin deficiency are consistent with previous observations [42, 43]. In the National Health and Nutrition Examination Survey (NHANES) survey of 1,770 elderly subjects in the USA, men had a significantly higher risk of cobalamin deficiency than women [44].

Comparable studies using holoTC as a marker to assess cobalamin status in the elderly people are rare [43, 45, 46]. In a Swiss cohort of unsupplemented subjects, mean holoTC concentrations were significantly lower in older subjects (60–69 years: 52.3 pmol/L, 70–79 years: 54.1 pmol/L, ≥ 80 years: 51.8 pmol/L) compared to our cohort with a mean concentration of 88.9 pmol/L [43]. There were significantly more women than men in our cohort, which means that the mean holoTC concentration is higher than in a “sex-balanced” cohort such as the Swiss cohort. However, even among the men in our cohort, the holoTC concentrations were significantly higher than those in the Swiss cohort. In an Irish cohort of elderly subjects (mean age 72.8 years, 35% men, not taking cobalamin supplements), the mean holoTC concentration (62.7 pmol/L) was also significantly lower compared than in our cohort [46]. The authors showed that the use of proton-pump inhibitors and the presence of atrophic gastritis led to significantly lower holoTC concentrations [46]. In our study, the use of proton-pump inhibitors was quite low (7%). Furthermore, male participants in our cohort had a higher prevalence of medical drug use, which may cause cobalamin malabsorption [13]. In addition, the frequency of medical drug use, especially non-steroidal anti-inflammatory drugs, may be crucial for mucosal damage and consequently reduced availability of food-bound cobalamin [47, 48].

Milk and dairy products and meat and meat products are food groups considered to be good sources of cobalamin. As expected, intake of milk and dairy products was significantly associated with holoTC concentrations and subjects with deficient cobalamin status consumed significantly less milk and dairy products than subjects with sufficient cobalamin status (data not shown). However, this finding cannot explain the overall good cobalamin status, because the subjects consumed less milk and dairy products (women: 162 g/day; men: 132 g/day) than the average German population aged 65–80 years (women: 210 g/day; men: 223 g/day) [16]. In contrast, meat consumption in our cohort (women: 77 g/day, men: 89 g/day) was much higher than in the German population (65–80 years, women: 46 g/day; men: 79 g/day). Surprisingly, no associations were found between holoTC concentrations and meat consumption, and in particular, the high meat consumption among male subjects in this cohort was contrasted with to the significantly higher prevalence of cobalamin deficiency in men.

### Folate status

In contrast to serum folate, which is subject to large fluctuations depending on acute dietary intake, RBC folate reflects long-term folate supply and is considered the most reliable marker of the folate status [49, 50]. We observed a predominantly sufficient folate status in the present cohort of elderly people. Comparison with the results of previous studies using the same tissue markers is limited by the fact that subjects in these studies mainly supplemented B vitamins. Folate fortification is not mandatory in Germany, but it is in many other countries. Pfeiffer et al. [44] evaluated RBC folate concentrations in older subjects (≥ 60 years) of the NHANES cohort before (1988–1994) and after the start of mandatory folate fortification in the USA (1999–2010). The prevalence of folate deficiency was similarly low before (2.1%) and after folate fortification (0.1%).

The adequate folate status in our cohort may be explained by the high intake of vegetables, which are considered to be a good source of folate. With a vegetable consumption of 255 g/day (women) and 222 g/day (men), the subjects in our cohort consumed almost twice the average of the German population aged 65–80 years (women: 128 g/day; men: 123 g/day) [16]. As expected, vegetable consumption was significantly associated with RBC folate concentration. Öhrvik et al. [51] also observed an association between vegetable consumption and RBC folate concentration in a cohort of adults (45–80 years, 46% women), 98% of whom had an adequate folate status.

The use of proton pump inhibitors and regular use of metformin have been described to negatively affect folate status [52]. In our population, the use of these drugs was very low or absent. Smoking [53], physical inactivity and a BMI ≥ 30 kg/m^2^ [52] also influence the folate status. However, our subjects were almost exclusively non-smokers, predominantly active and had an age-appropriate BMI. Taken together, these reasons may explain the overall sufficient folate status.

### Strengths and limitations

The strength of our study was its straightforward design with well-characterised subjects. In addition, we used state-of-the-art analytical parameters and methods to measure the vitamin D, folate, and cobalamin status. For example, 25-(OH)D concentrations were analysed by LC-MS/MS, which is considered as the gold standard method. Folate status was assessed using RBC folate, which is the most sensitive marker for assessing the body’s’ folate status. We also used holoTC, which is the first-line cobalamin marker in populations over 50 years of age [15].

The study has several limitations. The study has a relatively small sample size. As the study cohort consisted mainly of highly educated, active and health-conscious individuals who were willing to participate in a clinical study, the results cannot be extrapolated to the average community-dwelling elderly population in Germany, which does not have the same health-consciousness and physical activity.

The study was conducted during the summer season in Germany, reflecting a favourable situation concerning UVB-radiation and endogenous vitamin D synthesis. In addition, the study was conducted in the midst of the COVID-19 pandemic, when indoor gatherings were prohibited, creating conditions that make comparisons with other studies difficult.

The evaluation of nutrient intake data from food records generally has some bias. Alcohol intake was measured only by 3-day food records and not by food frequency questionnaires. Consumption of fortified products was not asked separately in a validated questionnaire. However, in Germany, food is only occasionally fortified with folic acid compared to other countries, and no significant influence of fortified products can be assumed. When comparing the food group intakes of our population with the average German population [16], it should be noted that the representative reference data were collected over a period of 4 weeks using different survey methods (e.g. food frequency questionnaires, dietary history interviews, weighing records), whereas our data were collected by 3-day food recalls only.

The metabolic markers homocysteine, methylmalonic acid and the aggregated marker 4cB12 are used in clinical trials to assess the cobalamin and folate status [54]. However, these markers may be influenced by impaired renal function or deficiencies of other B vitamins involved in homocysteine metabolism. Finally, we don’t have information on the prevalence of infection with *Helicobacter pylori* infection or atrophic gastritis in our cohort, which may predict dietary cobalamin malabsorption. However, cobalamin deficiency was quite low and the prevalence of atrophic gastritis is rather high in Germany [55].

## Conclusion

Overall, we observed a low prevalence of vitamin D, cobalamin and folate deficiency in a cohort of individuals aged ≥ 70 years, characterised by high levels of education, physical activity, and health awareness. However, despite a healthy and active lifestyle, a significant proportion of male subjects did not achieve adequate concentrations of holoTC or RBC folate. The latter result must be treated with caution as the number of men in the study cohort was very small. The consolidation of these findings needs to be investigated in further studies, which should explicitly focus on subjects of advanced age > 80 years. In addition, studies are needed to identify lifestyle and dietary patterns that may predict adequate nutrient status in older people and ensure healthy ageing.

### Electronic supplementary material

Below is the link to the electronic supplementary material.

Supplementary Material 1

## Data Availability

The datasets used and/or analyzed during the current study available from the corresponding author on reasonable request.
