# Supplementary material for: Folate, vitamin B12 and vitamin D status in healthy and active home-dwelling people over 70 years
Source: BMC Geriatr. 2023 Oct 18;23:673. doi: 10.1186/s12877-023-04391-2 (PMC10585793; doi:10.1186/s12877-023-04391-2)
Supplement: Supplementary file 1 — Supplementary Material 1 [file 12877_2023_4391_MOESM1_ESM.docx]

**Folate, Vitamin B12 and vitamin D status in healthy and active home-dwelling people over 70 years**

Felix Kerlikowsky 1; Jan Philipp Schuchardt 1; Andreas Hahn1

1 Institute of Food Science and Human Nutrition, Leibniz University Hannover, Germany

Correspondence: Andreas Hahn

Leibniz University Hannover

Institute of Food Science and Human Nutrition

30167 Hannover, Germany

Email: hahn@nutrition.uni-hannover.de

Tel.: +49 511 762 2987

Fax: +49 511 762 5729

Keywords: Nutrient status, HoloTC, RBC Folate, ageing

**Supplementary material**

**Table S1: Linear regression models to examine the association of age and vitamin status markers.**

|  | **Beta-coeff.** | **Modell 1**  **p-value** | **Modell 2**  **p-value** |
| --- | --- | --- | --- |
| **HoloTC** | -0.134 | 0.118 | 0.161 |
| **RBC folate** | 0.001 | 0.988 | 0.979 |
| **25-(OH)D** | -0.006 | 0.075 | 0.101 |

Modell 1: unadjusted**;** Modell 2: adjusted for gender, body weight, BMI, WC.

**Table S2: Anthropometric and vitamin status markers in different age groups.**

|  | **Age groups** | | |  |
| --- | --- | --- | --- | --- |
|  | **70-74 years**  n=61 | **75-80 years**  n=51 | **≥80 years**  n=22 | **p-value**  (f vs. m) |
| **Anthropometric marker** | **Mean±SD** | **Mean±SD** | **Mean±SD** |  |
| **Weight** [kg] | 68.6±14.9 | 71.1±12.2 | 73.5±14.4 | 0.248† |
| **Body mass index** [kg/m^2^] | 25.3±5.2 | 25.6±3.4 | 26.7±5.1 | 0.446† |
| **Waist/Hip-Ratio** | 0.89±0.08 | 0.91±0.10 | 0.94±0.07 | 0.052† |
| **Systolic blood pressure** [mmHg] | 141±15.2 | 146±17.6 | 149±17.1 | 0.142† |
| **Diastolic blood pressure** [mmHg] | 84.6±11.0 | 84.8±10.2 | 84.1±13.3 | 0.943† |
| **Vitamin status markers** | **Mean±SD**  n (%) | **Mean±SD**  n (%) | **Mean±SD**  n (%) |  |
| **HoloTC** [pmol/L]  Deficient [<50 pmol/L] | 91.7±33.4  5 (8) | 89.2±34.1  6 (11) | 80.7±33.6  5 (23) | 0.375†  0.197‡ |
| **RBC folate** [nmol/L]  Deficient [<570 nmol/L] | 800±201  5 (8) | 860±250  3 (6) | 842±312  5 (23) | 0.526†  0.072‡ |
| **25-(OH)D** [nmol/L]  Deficieny [≤50 nmol/L]  Insufficieny [52.5-72.5 nmol/L]  Sufficiency [≥72.5 nmol/L] | 85.6±30.0  7 (12)  15 (24)  39 (64) | 86.1±20.1  3 (6)  11 (22)  37 (73) | 81.5±27.0  2 (9)  8 (36)  12 (54) | 0.590†  0.536‡ |

† One-way ANOVATest; ‡ chi-squared test.

**Table S3: Linear regression models to examine the association of food group intake and vitamin status markers.**

|  | **Beta-coeff.** | **Model 1**  **p-value** | **Model 2**  **p-value** |  |
| --- | --- | --- | --- | --- |
| **HoloTC** | | | | |
| **Milk and dairy products** | 0.235 | **0.008** | **0.006** |  |
| **Meat, eggs, meat products** | 0.015 | 0.300 | 0.914 |  |
| **RBC folate** | | | | |
| **Fruits** | 0.001 | 0.865 | 0.878 |  |
| **Vegetables** | 0.001 | **0.014** | **0.038** |  |
| **Seed and nuts** | 0.001 | 0.059 | 0.087 |  |
| **Grains and bread** | -0.001 | 0.285 | 0.441 |  |
| **25-(OH)D** | | | | |
| **Fish** | 0.003 | 0.541 | 0.769 |  |

Model 1: unadjusted**;** Model 2: adjusted for gender, body weight, BMI, WC.

**Table S4: Nutrient intake in different sexes.**

|  | **Total**  n=134 | **Female**  n=97 | **Male**  n=37 | **p-value**  (f vs. m) |
| --- | --- | --- | --- | --- |
|  | **Mean±SD** | **Mean±SD** | **Mean±SD** |  |
| **Total energy** [kcal/day] | 1800±596 | 1723±593 | 2006±563 | **0.004†** |
| **Total carbohydrate** [g/day] | 179±61.4 | 198±61.6 | 171±60.0 | **0.013†** |
| **Total fat** [g/day] | 74.3±32.3 | 70.6±30.9 | 84.6±34.2 | **0.008†** |
| **Total protein** [g/day] | 68.4±25.8 | 66.4±26.8 | 74.1±22.6 | **0.043†** |
| **Carbohydrates** [En%] | 40.9±7.5 | 41.0±7.6 | 40.3±7.3 | 0.384† |
| **Protein** [En%] | 15.7±3.8 | 15.9±3.5 | 15.2±2.9 | 0.458† |
| **Fat** [En%] | 37.0±6.6 | 36.7±6.2 | 37.6±7.5 | 0.513† |
| **Total alcohol** [g/day] | 9.6±12.9 | 7.8±11.8 | 14.2±14.8 | **0.031†** |
| **Total fiber** [g/day] | 21.3±8.1 | 21.5±7.9 | 20.7±8.7 | 0.378† |

† student’s T-test.
